# IoT-Blockchain Enabled Optimized Provenance System for Food Industry 4.0 Using Advanced Deep Learning

**DOI:** 10.3390/s20102990

**Published:** 2020-05-25

**Authors:** Prince Waqas Khan, Yung-Cheol Byun, Namje Park

**Affiliations:** 1 Department of Computer Engineering, Jeju National University, Jeju City 63243, Korea; princewaqas12@hotmail.com; 2 Department of Computer Education, Teachers College, Jeju National University, Jeju City 63243, Korea; namjepark@jejunu.ac.kr

**Keywords:** livestock, internet of things, blockchain, advanced deep learning, industry 4.0, provenance

## Abstract

Agriculture and livestock play a vital role in social and economic stability. Food safety and transparency in the food supply chain are a significant concern for many people. Internet of Things (IoT) and blockchain are gaining attention due to their success in versatile applications. They generate a large amount of data that can be optimized and used efficiently by advanced deep learning (ADL) techniques. The importance of such innovations from the viewpoint of supply chain management is significant in different processes such as for broadened visibility, provenance, digitalization, disintermediation, and smart contracts. This article takes the secure IoT–blockchain data of Industry 4.0 in the food sector as a research object. Using ADL techniques, we propose a hybrid model based on recurrent neural networks (RNN). Therefore, we used long short-term memory (LSTM) and gated recurrent units (GRU) as a prediction model and genetic algorithm (GA) optimization jointly to optimize the parameters of the hybrid model. We select the optimal training parameters by GA and finally cascade LSTM with GRU. We evaluated the performance of the proposed system for a different number of users. This paper aims to help supply chain practitioners to take advantage of the state-of-the-art technologies; it will also help the industry to make policies according to the predictions of ADL.

## 1. Introduction

Industry 4.0 incorporates wireless technology, sensors, and smart machines to create a wholly connected enterprise. The Industrial Internet of Things (IIoT) is considered to be a significant component of Industry 4.0; it collects information about processes, equipment performance, supplies, and orders, and using big data, it aggregates information from suppliers, manufacturers, and customers. Food safety is a significant concern in the modern world; governments need to quickly formulate policies and take various measures to strengthen the management of the safe production of agricultural products, including identification and tracking. Meat products are major components of human food along with agricultural products. Agriculture and livestock sectors play roles in meeting the hunger requirements of the rapidly increasing world population. The green agriculture and livestock revolution led to general increases in crop yields and meat consumption.

People not only want to eat, but they also wish to eat nutritious meals. According to the World Food Program, malnutrition is the leading cause of death of 45% of children under five years of age, and 2 billion people suffer from deficiencies of vitamin and minerals [1]. Many companies and people are working to improve the quality and quantity of food products [2,3,4,5]. Precision agriculture is a main focus of research in the 21st century; it is revolutionizing the agriculture industry by introducing several impressive technologies [6]. The emergence of information and communications technology (ICT)-based techniques is having a significant impact on the yields of crops; now, farmers can remotely monitor the conditions of their farms and can control their equipment remotely using a smartphone.

Farmers widely appreciate the use of unmanned aerial vehicles (UAV) to capture real-time data regarding crop health and to detect weeds [7]. ICT has helped us to efficiently use irrigation and water management systems for agriculture. It gives access to soil moisture deficit data and weather reports through online irrigation scheduling and data regarding the soil pH of agricultural areas. It allows the high-quality cultivation of orchards with the help of agricultural robots [8]. The use of information and communications technologies in the food sector has a significant impact; one such technology which is widely recognized for its transparency in the food supply chain is blockchain.

**Figure 1:**
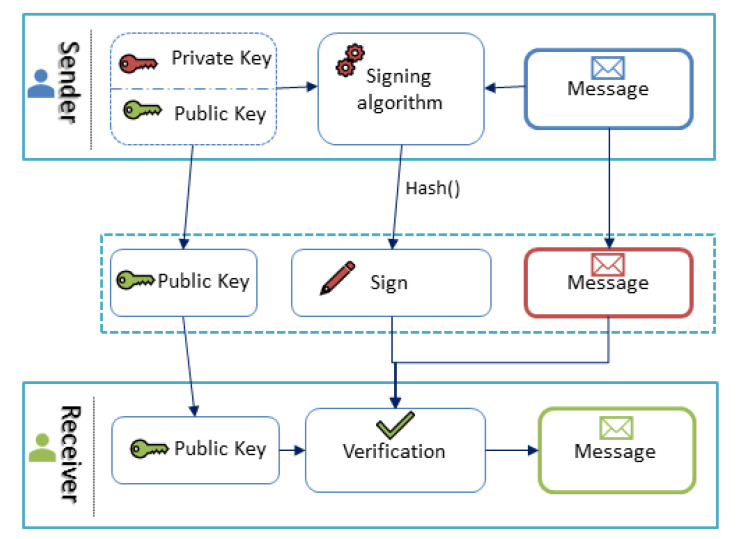
Cryptography scheme for transactions in a blockchain.

Blockchain was initially introduced for bitcoin [9]. Bitcoin is the latest innovation in economic computing, allowing customers to exchange money in a cheap reliable, and fast way using the internet. Blockchain has now emerged as the ultimate solution to many issues, such as logistical [10] and cost issues [11]. The Internet of Things (IoT) is also helping to connect devices and sensors, and the combination of IoT and blockchain is proving to be a time and money-saving approach—one which also generates a large amount of data. Analyzing these data using advanced deep learning techniques (ADL) helps manufacturers to make informed decisions [12]. However, only a small percent of manufacturers in the food and beverage industry use the IIoT and smart manufacturing. Blockchain-enabled IIoT systems can be optimized using deep reinforcement learning approaches [13]. Blockchain helps Industry 4.0 to secure sensor data [14], as the data within the blockchain are cryptographically encrypted. This makes blockchain a secure platform which can be trusted. Every user has two keys—public and private. Figure 1 shows a cryptographic scheme for message transactions in a blockchain. While transmitting the data, the public key is used to encrypt a message by the sender, and the person at the receiving end who has that specific public key can decrypt and read the message.

Artificial intelligence (AI) can be used to interpret data and provide the next steps in the manufacturing process if something goes wrong. We can even automate suggestions regarding how to fix an issue [15]. We can also use natural language processing to monitor our suppliers across borders with AI in the supply chain. AI reduces the time spent on manual labor, freeing employees to make more valuable contributions to a business. AI allows us to make better, more informed decisions based on prior historical data. AI provides critical alerts and recommendations for when things go wrong; it also helps us to monitor suppliers in other regions of the world to prevent the problem proactively. It simplifies supply chain relationship management and helps us to optimize our supply chain processes to increase customer satisfaction and generate more profit for the business.

Blockchain’s ability to trace a good through the entire supply chain is one of its most useful applications [16]. The fact that blockchain’s distributed ledger is almost impossible to change serves as the perfect tool for maintaining a comprehensive record of the exchange of ownership that occurs throughout supply chains. The advantages of blockchain technology (BCT) have attracted the scientific community to determine its possible uses in the field of the supply chain. This technology can control and monitor risk mitigation efforts and strengthen the supply chain, and it could prove useful in preventing security breaches.

Dairy products such as meat, milk, yogurt, butter, and cheese play a vital role in meeting the food requirements of humans. Meat is an essential part of food; since the very beginning of humanity, meat has been a significant source of food. Due to high demand, it has been shown that many companies overlook the safety of meat, and have distributed old and rotten meat to the consumers [17,18]. Recently, scandals have emerged around the world regarding replaced, stale and tainted meat, including the famous horse-meat scandal in Britain [19], tainted meat in Brazil [20], the expired meat scandal in China [20], and donkey meat in Pakistan [21]. To highlight the importance of meat traceability, Sander et al. [22] investigated different perspectives of the meat supply chain; they also performed a survey for this purpose and evaluated how BCT could help in the transparency and traceability of the meat supply chain system. The focus of the author was to determine consumer views about BCT in the supply chain for traceability and transparency. The author used different research methods to check the efficiency of the transparency and traceability of this system. They used an online survey by setting up a questionnaire and conducted interviews with retails managers, government officials, and third-party transparency service providers. With transparency and traceability systems, people will assume better quality, which will increase their purchasing decision power. Although transparency and traceability systems will help meat suppliers to achieve a better reputation, the costs will be increased, which can be an issue for customers.

In this work, we propose a combination of these state of the art technologies. We combine the IoT and blockchain with ADL. We propose a hybrid model based on recurrent neural networks (RNN) algorithms to forecast the supply and demand of food, using long short-term memory (LSTM) and gated recurrent units (GRU) as a prediction model and the Genetic Algorithm (GA) optimization jointly to optimize the parameters of the hybrid model. In the wholesale and trading centers for meat products, hundreds of tons of meat flow from various farmers’ markets into the urban area every day. To ensure safety, the proposed system establishes a blockchain-based digital ledger for each food product, and users can quickly learn about production information, quality information, market information, price information, etc. through the system. The blockchain records the origin, transaction time, and inspection and quarantine information of each product that enters the market. The end-user only needs to enter the number of transactions or scan the barcode, and they will be able to trace the origin of the food. Due to the involvement of IoT, customers will get access to the food’s temperature history; the functional modules of the proposed system also include administrative modules for farmers and the warehouse owner.

This paper starts with the study of recent advances in IoT and blockchain in the food industry. In Section 2, we describe the current research on how blockchain and IoT can transform the food industry, and review the most recent developments in this practice. In Section 3, we present the outline of the design of the IoT and blockchain-enabled system model. In Section 4, we explain our proposed solution, which is based on a private blockchain platform and hybrid deep learning model. Section 5 comprises an evaluation of the working of our proposed model. We close by highlighting our contribution to the literature and the implications of the proposed model in Section 6, followed by a discussion of specific confinements involved in the implementation of such systems. We summarize our work in Section 7.

## 2. IoT and Blockchain in Food Industry

The provenance system of agricultural products is essential to ensure food safety, but there are numerous and physically dispersed stakeholders involved in the agricultural supply chain process, including the growers of a specific product, farmhouses, and the sellers of those products, which are large in number and not connected. Every company involved in the supply chain of agriculture products has its own data recording systems; therefore, it is difficult to achieve a central tracking system for information because of the incompatibility among software or data structures. Table 1 provides a comparison of existing IoT or blockchain-based supply chain solutions. Lin et al. [23] proposed an ecosystem for smart agriculture based on BCT and the Internet of Things (IoT). According to this system, all the stakeholders can use their smartphones to input data into this system, and it also collects the data from IoT devices itself without the intervention of any other person. To ensure the effectiveness of this model, they used the virtual trusted trade BCT Network Cloud Platform as a core of their architecture. According to this model, two types of transactions are performed: one is enterprise resource planning, which includes trade, logistics, delivery, warehousing information; and the second includes data from IoT devices, such as the air temperature, air humidity, soil PH, soil nutrition, ground moisture, etc. This model is based on BCT, meaning that all the data communicates through nodes after hashing and digital signatures. Such systems can resolve the food safety issues of the end consumers. The combination of BCT and IoT technologies can be useful in the building of a trusted, self-organized, open, and ecological smart agriculture system.

IoT and Blockchain technologies (BCT) can transform the food industry in three main areas:

- Provenance;
- Payments;
- Management.

**Figure 2:**
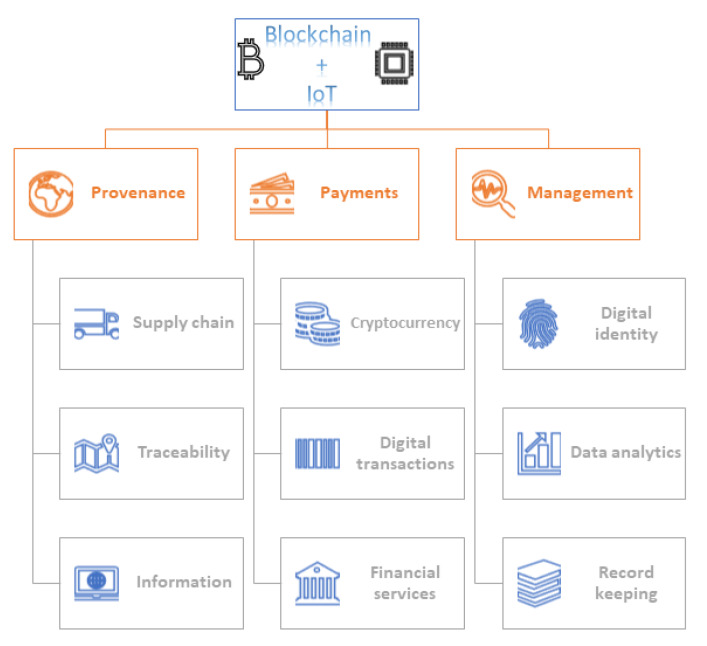
Applications of IoT and blockchain in the food industry.

Figure 2 summarizes the significant demands of IoT and blockchain in the food industry. It introduces the essential features of different sections. The provenance section comprises the supply chain, traceability, and information system; the payments section focuses on cryptocurrency, digital transactions, and financial services; and the management section includes digital identity, data analytics, and record keeping.

### 2.1. Provenance

Provenance and radical transparency is the key to food safety and consumer trust. With the help of BCT, farmers can make mobile payments and credits; also, the transaction fees will be decreased. The real-time management of supply chain transactions and financing will prove to be of enormous benefit in the agricultural and food sector. Using blockchain, a more direct link can be established between supplier and retailer to make sure that farmers receive fair payment for their products, and retailers can confirm that they are getting what they have paid for [33]. The digital tokens based on BCT can be exchanged for fertilizer for small farmers. These tokens cannot be misused or imitated since they are on a blockchain and can be traced to make sure that the government-allocated funds create the maximum impact in the areas for which they are intended. Blockchain-based agriculture can be very rich in terms of information for the end-users. For example, a customer buys a pack of cereal from the market near their house; they can trace it back from the warehouse to the farms where it was grown or even further back to the shop from which the seeds were purchased. All they have to do is to scan the barcode by using their smartphone [34], which will show all transaction data related to it, including the arrival date of this packet into the market, from which farm the cereals were produced, on which day and time it was created, the ID of the farm owner, the ID of the staff who collected the plants, collecting device information, packaging information, all the temperature and other environmental data for the plant’s production, process, logistics, storage, etc. The blockchain system can verify all of this information without human intervention.

To use the BCT in agriculture is a significant issue because it is complicated to automate storage and then to obtain the hash data. Xie et al. [35] proposed a storage scheme for the tracking of agricultural products to address this problem. They designed a secured data storage scheme to track agricultural products based on BCT with a double-chain storage structure. They not only implemented the plan but also built a practical application to verify its correctness. According to their proposed system, agricultural products are bound with IoT sensors; the sensor obtains the data of the agricultural products and uploads them to the server in real-time. A double-chain storage structure is used simultaneously to store the data in the blockchain automatically; the system can also efficiently look into the data and provide them to the upper application. The system structure contains three layers: the application layer, which is used to view and monitor the tracking of nodes; the data storage layer, which consists of a blockchain system based on Ethereum; and the third layer, which is the sensing layer, using sensors mounted on different devices and in different locations. This scheme is based on a double chain structure, so a parent transaction hash is added into the database; in this way, all the data of the identity can be obtained only using the last transaction hash, and data will not have been changed. To improve the storage scheme, the filtering of data is done in tracking applications. For this, a non-normal data judgment strategy is added, and through the experiments, the authors observed that this scheme provides higher data security as compared to non-chain mode; on the other hand, the of querying the data is slow.

Tian et al. [36] also proposed a traceability model for the supply chain of food in the context of China. This system was based on RFID and BCT. According to the author, this system could prove beneficial to rule out fake products from the market. Nevertheless, on the other hand, its cost is high, it is immature, and more work is required. This system covers the whole process of data gathering and information management for every link in the agri-food supply chain, which tracks and registers the quality and safety of the agri-food “from farm to fork”.

### 2.2. Payments

ICT agricultural systems with a BCT foundation are localized and unchangeable management systems of records. Tseng et al. [37] proposed a system model of an ICT e-agricultural system with a blockchain infrastructure, in which BCT is used to store the data of water quality monitoring. This system can help to monitor the amount of water which is suitable to obtain a high yield. The author also proposed an evaluation tool; this proposed prototype ICT e-agriculture infrastructure, built at National Taiwan University, was used to monitor the quality of irrigation water-related data collected by remote sensors at various farm locations and used the GCOIN blockchain system. GCOIN is a type of private blockchain which was developed by a professor of the National Taiwan University as a project in 2016.

In the agri-food sector, market information is non-symmetric, and there is no guarantee of market fairness. To eliminate these issues, Mao et al. [27] proposed a food trade mechanism based on an alliance chain. To design a new architecture, the authors used consortium BCT; this helps to protect the safety of the transactions. Due to the involvement of different roles, authentications, and permissions for food transactions in the agri-food supply chain, ensuring the safety of transactions is very challenging; this technology is beneficial in meeting these challenges. Another issue is the improvement of transaction efficiency and assisting users in finding suitable transactions. Regarding this issue, the improved practical byzantine fault tolerance (iPBFT) algorithm was designed, and to eliminate competition, the authors suggested using an online double auction mechanism. This newly developed system was named the food trading system with consortium blockchain (FTSCON). In this system, there are two nodes: the user node and scheduling node. The user node can be a buyer or seller node, depending upon the situation. In addition, scheduling nodes are used to authorize and to verify the transaction data of the system. Before recording a transaction in this chain, consensus processing is applied to verify the data, known as a consortium blockchain. This includes a three-part block, which contains the transaction data, mechanism of consensus, and smart contract. This system was made with the Ethereum architecture, which includes consortium blockchain.

### 2.3. Management

Hua et al. [38] proposed a platform design to resolve two critical issues in agricultural traceability systems: the first is the credibility of the data, and the second is the difficulty of integrating the subsystems of each company involved in this traceability system. According to the designed platform, a distributed peer-to-peer platform is used to overcome the problem of data credibility. Once the data enter into the distributed network and a consensus is formed, it can no longer be modified. Secondly, to overcome the issue of a subsystem consolidation-designed platform, an open data-sharing platform is used. However, this system has a loophole: this system is a free data-sharing platform, meaning that data will be visible to all stakeholders. However, most companies do not want to share their data with anyone. Therefore, to overcome this problem, every stakeholder has to choose a different license. Leng et al. [39] proposed an agricultural supply chain system using the concept of the public blockchain, which is based on a double chain architecture. According to this concept, BCT could be public or private based on the rights to record the data. In public blockchains, the information privacy of users cannot be protected, and the transaction speed is low, whereas in the private blockchain, the speed of transactions and consensus is fast. The authors designed a public blockchain for agricultural business.

At present, China is a massive market for agriculture. It has to feed 22% of the world population. From the perspective of China, Tse et al. [40] conducted the first in-depth analysis of blockchain application in food supply information security. In their paper, they used theories from different fields of science, including IT, management, system, and empirical research methods. According to their study, by using BCT, we can achieve the aim of replacing the paper tracking system, and we could have a permanent, non-tempered record for every transaction. They used a PEST analysis analytical model, which analyzes political factors, economic factors, societal factors, and technology factors. According to this analysis, the government has the critical responsibility for food safety, so it should create an environment in which food supply information systems could grow, and regulatory authorities must look after those systems. In the society analysis, the author said that people currently want more information about what they are buying; thus, every step of the food supply chain must be accessible to the consumers. The author suggested that this system should be developed in China, and forceful actions should be taken.

Although many models and schemes have been proposed for supply chain models based on BCT, no works have considered the supply chain from the view of hazard analysis and critical control points. Tian et al. [41] proposed a food supply chain traceability system with the combination of BCT and Internet of Things based on hazard analysis. This work mainly focuses on risk management and its prevention from food safety points. They divide the food supply chain into five links: a production link, where the environment of the crop should be assessed; the processing link, which requires the assessment of processing equipment; the warehousing link, which depends upon the maintenance of cold-chain equipment; the distribution link, which requires the proper maintenance of transporting trucks or vehicles; and finally, the e-retail link, where all retail management practices are evaluated according to good working practices. This traceability system is a decentralized distributed system. This system uses different IoT technologies to collect data such as RFID, GPS, and wireless sensor networks (WSNs). To store and manage data, BigchainDB is used because it has high throughput, low latency, and powerful query functionality. This system can be helpful in rebuilding the confidence of consumers of the food industry.

In the existing supply chain of agriculture, the relationships between members are not very strong. In work done by Casado et al. [42], a model to strengthen this relationship and to ensure the right of information of customers was proposed. The European Regional Development Fund supported this work. This model involves BCT, smart contracts, and a multi-agent system to coordinate the tracking of food in the agriculture supply chain. Blockchain is currently used in many applications. This is a computerized system, which consists of multiple intelligent agents who interact with each other. The use of smart contracts in the blockchain to manage the entire supply chain makes the process more efficient.

## 3. System Model

**Figure 3:**
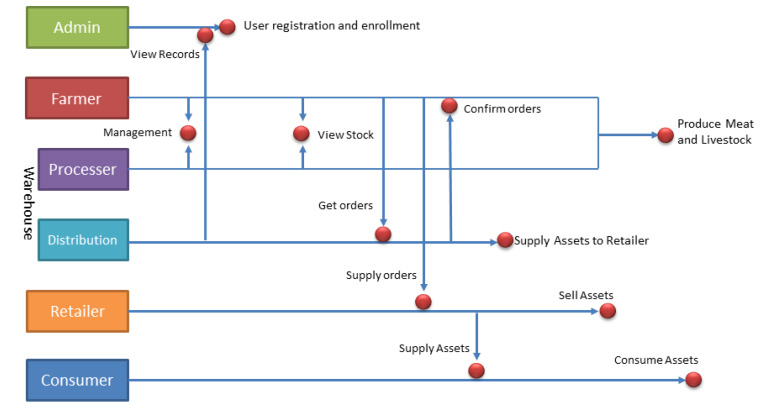
Designed system scenario.

Many participants are involved in a food supply chain, but we can categorize them into four main divisions: farmer, warehouse, retailer, and consumer. The warehouse also consists of two subunits: the meat processor and distribution unit. Our proposed food supply chain system is designed to take full advantage of the state-of-the-art technologies such as the Internet of Things, blockchain, and artificial intelligence. Figure 3 demonstrates the designed system scenario of the proposed IoT and blockchain-enabled intelligent provenance system.

The administrators can enroll new participants and can see the records. Farm owners can deal with the warehouse for the management of livestock. The farmer is responsible for identifying each animal with an ear tag equipped with an electronic label at the farmhouse; they are also responsible for uploading the data to the blockchain through their mobile device or computer. The warehouse updates the records of the total assets present at the slaughterhouse through the weighing platform, attaches a hook label for raw meat during the slaughter process, and transfers all types of data to the blockchain. In the processing of divided meat, each part of the split meat is uniquely identified by barcode technology, and a related information tracking system is established to record the production process of the divided meat. All kinds of data are aggregated to the blockchain server. In the warehouse, there are two central units: processing and distribution. The processing unit deals with the cutting of meat and packaging, whereas the distribution unit is responsible for supplying the assets to the retailer. In the case of live animals, the farmer can directly provide the animal to the retailer. The last—and one of the most significant—parts of the system is the end consumer, who buys the assets from the retailer and consumes it. The consumers could be restaurant owners or individual customers. Finally, the blockchain can be used to query meat products through mobile tags or serial numbers, to establish a complete management system for livestock breeding and meat product production and sales.

## 4. Proposed Solution

We have proposed an IoT and blockchain-enabled optimized provenance system for Industry 4.0 in the food sector using advanced deep learning. We have used Hyperledger Fabric as a blockchain for our proposed method; Hyperledger Fabric allows the network manager to manage a private blockchain in a user-friendly manner.

**Figure 4:**
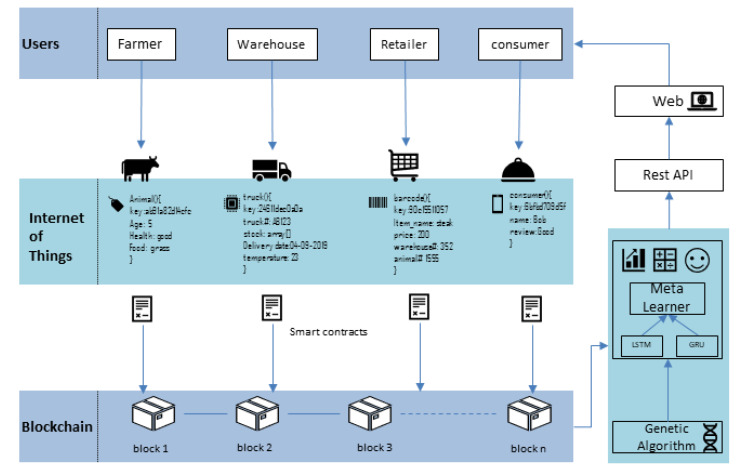
IoT–blockchain-enabled intelligent provenance system.

Figure 4 demonstrates the architecture of the IoT–Blockchain-enabled intelligent provenance system. In our proposed method, we have taken farm owners as participants and allowed them to establish a file of meat products for each animal. The farmer can create personnel files and duty record files for each section. They can also use smart contracts and event notification functions for the upper and lower limits of inventory information. The second participant is the processing unit of the warehouse; in the production and processing link, the data identified by the ear tag in the breeding unit are transferred to the production and processing unit. The processing unit is responsible for the information regarding different nodes of the production and processing, which is collected according to the management standards and specifications and uniquely identified through the barcodes. Then, the data are transferred to the distribution unit. The distribution unit is responsible for transferring assets to the retailers. During the transportation of the goods, this information is written on the pallet of the delivered goods or the barcode of the packaging box according to the specified standards of the origin, quantity, quality, grade, and so on. While taking full advantage of IoT, each delivery truck will have temperature sensors and GPS for tracking. When the goods are transported to the designated retailer or designated distribution point of the wholesale market or shopping mall, the information regarding the products stored in the labels of the packaging boxes must be read first and uploaded to the system. For warehousing and logistics distribution management, a traceability system is established in the production and processing and store supply chain through barcodes. In logistics, the goods information is recorded on the label of the pallet or goods box. In this way, the barcode system can house the location, identity, storage and transportation history, destination, expiration date, and other useful information of the boxes on the pallet or even individual items. The barcode system can provide detailed data for the actual goods in the supply chain and establish a physical connection between the products and their complete identity. Users can easily access these completely reliable goods data, and through the efficient data collection system of the barcode, the warehouse logistics information can be fed back to the production and processing stage in time to guide the production. At the point of sale (PoS), the end consumer must be informed of the origin of the meat product; thus, retail labels on packaged meat products must have human-readable information, and non-packaged meat products must provide relevant information in other ways.

### 4.1. Private Blockchain Platform

The blockchain refers to distributed data technology. It is a method for recording data using cryptographic hash functions; in the field of computer network security, this is a critical feature. To secure a network, different authentications methods are implied. Usually, a server is used to authenticate the users, but there is no centralized system in the blockchain because it is a peer-to-peer network. Therefore, to verify the users in these networks, digital signature technology in encryption algorithms is used. In this type of encryption, a pair of keys is used: one is public, and the other is private. The private key can decrypt the data encrypted by the public key, and vice versa. Users in the blockchain system use an asymmetric encryption method to indicate their identity; in this way, the user keeps ther private key and distributes the public key to others. Their private key signs the information sent by the user, and the other users use the corresponding public key to verify the authenticity of the data. Blockchain also provides an essential feature of a smart contract to automate the trade between two parties without the involvement of an intermediary [43]. The smart contract is the soft form of a contract written in the programming language.

**Figure 5:**
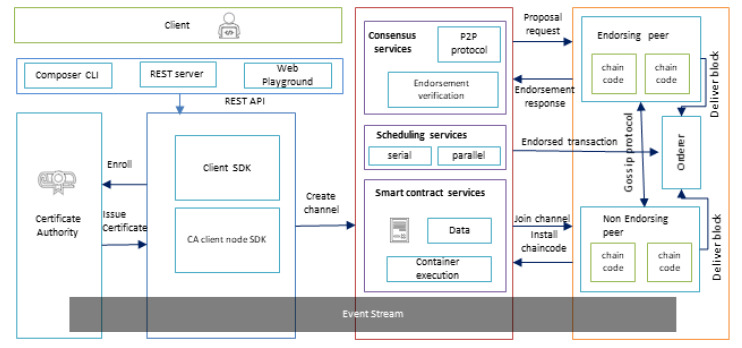
Architecture diagram of a private blockchain.

In this article, Hyperledger Fabric is used as a private blockchain platform. It is an open-source platform developed by IBM. Hyperledger composer is used as a tool to build and deploy the private blockchain [44]. It allows the users to write smart contracts and define participants and assets using the command-line interface (CLI). Figure 5 presents the general architecture of the private blockchain platform. In a private blockchain network, only authorized clients can interact with the system. Users interact with the Hyperledger network with the REST server [45]. Every user is enrolled by the certificate authority (CA). Hyperledger Fabric contains different types of services, such as consensus services, scheduling services, and smart contract services. There are also three kinds of peers: the endorsing peer, non-endorsing peer, and the orderer. This network consists of different nodes, and every node holds a copy of the immutable chain. This chain consists of a different block, and every block consists of the hash value, encrypted data, and timestamp.

**Figure 6:**
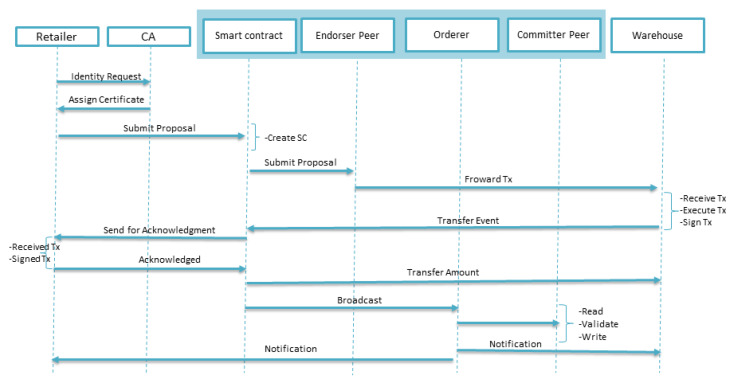
Transaction processes in the blockchain.

Figure 6 shows the sequence diagram of a transaction between the retailer and warehouse within the blockchain network. Hyperledger provides the certificate authority (CA), which is responsible for registering new nodes and issuing certificates. The retailer first needs to be registered by CA; only a registered participant can initiate a transaction. The retailer will submit a proposal to the smart contract, which will be forwarded to the warehouse by endorser peer. The warehouse will execute the transaction and generate the completion of the transfer event. A smart contract, after checking the predefined rules, asks the client to acknowledge. On successful acknowledgment, the smart contract will release the amount to the warehouse and broadcast this to the orderer, which will forward this for validation and writing purposes. In the end, a notification will be sent to all parties on the successful completion of the transaction.

### 4.2. Endpoint Security

**Figure 7:**
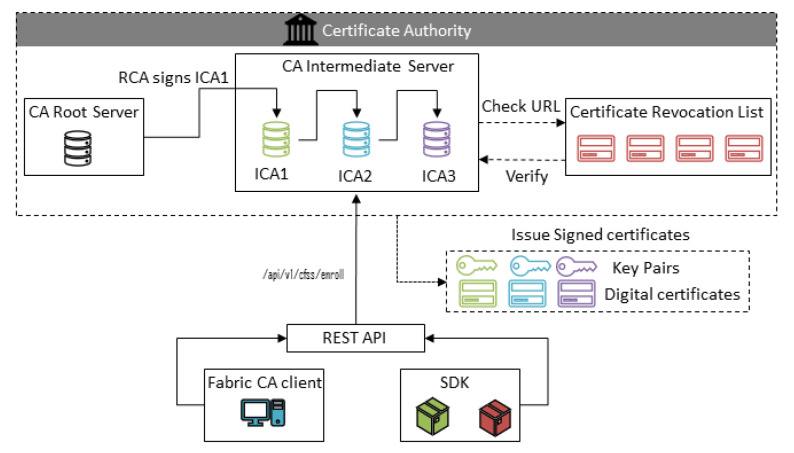
Identity management system for endpoint security.

Endpoint security deals with the protection of endpoints or end-user nodes. Endpoints act as access points to the blockchain network. Malicious parties can use these points; therefore, in a secure supply chain, securing endpoints is important. Hyperledger Fabric provides digital certificates to the end nodes to secure the network. All sorts of nodes of the network, including admin, peers, orderers, and clients, need certificates to interact with the network. Figure 7 depicts the identity management system for endpoint security in the Hyperledger blockchain. CA assigns the certificate to the nodes; one central root certificate authority (RCA) can assign the certificate to an intermediate certificate authority (ICA), and each ICA can further assign the certificates to another ICA and nodes as well. A certificate revocation list (CRL) is also present, which consists of malicious or revoked certificates. Before assigning the certificate to the nodes, the CA checks it against the CRL. Nodes can interact with CA through the fabric CA client or software development kit (SDK).

### 4.3. Integration of IoT with Blockchain

**Figure 8:**
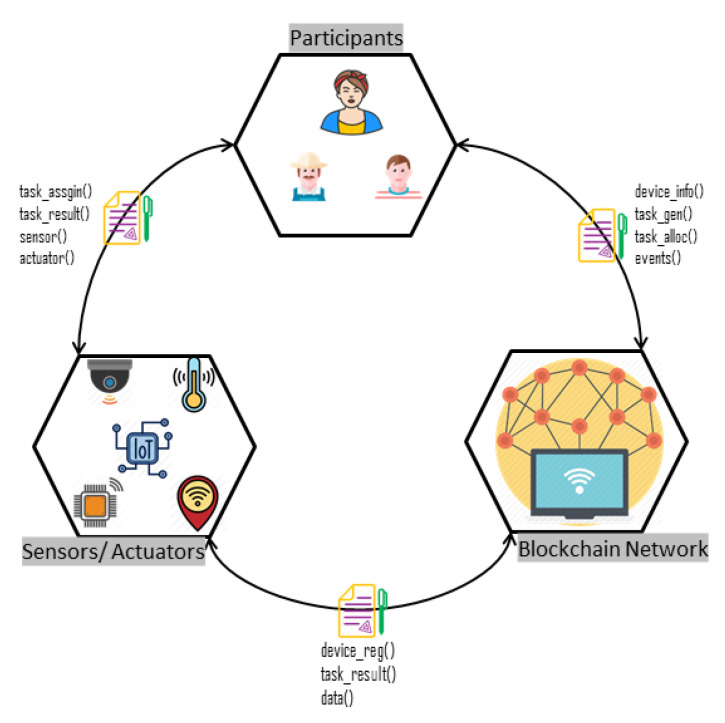
Integration scenarios of IoT with blockchain.

According to the goal of the Internet of Things (IoT), conventional devices become smart and autonomous. Blockchain has emerged as a critical technology that will transform how we share information. Building trust in distributed environments without the need for authorities is a technological advancement that has the potential to change many industries, including the IoT. The integration of IoT with blockchain in the food supply can be beneficial in automating tasks and saving time. Figure 8 describes the integration scenarios of IoT with the blockchain and the interaction of participants with them.

The functions that are involved in the integration process are device registration, task generation, task allocation, and task verification [46]. The first step is to register the IoT device with the network; the membership service provider will register the IoT devices, and each device will receive a unique identity to communicate with the system. The second step is to generate the tasks for the IoT devices; device owners can specify the required task information, which can include a task ID, device ID, device data, and participant information, and they will submit a transaction proposal to the smart contract. The nodes of the blockchain network execute the transaction proposal and append the transaction in the ledger. An event is emitted from these nodes to inform the device of whether the transaction succeeded or failed.

The next step is to allocate the task from the sensors or actuators. For this purpose, the device owner has to again submit a proposal to the smart contract with sensing task information; if the network permits the request, then an event is emitted from these nodes to inform the device owner that they can assign the sensing or actuating task to the sensor or actuators. Then, an event is emitted from these nodes to notify the device owner that the actuator status is changed. The final step is task verification: smart contracts are used to verify the tasks according to the predefined set of rules. These rules are defined at the time of building the network. The sensor state can be updated using Algorithm 1. The user needs a sensor ID to obtain the data of a specific sensor or actuator. The algorithm starts with initializing the asset and device ID values. Here, t-sensor represents the list of sensors. The algorithm will check if the transaction state for a specific sensor is new; then, it will change the state of the sensor, and it will also enable the transaction. After that, it will update the sensor registry and generate an event to notify the user that the state has been updated.
**Algorithm 1** Pseudo code for sensor state transaction.**Ensure:** Initialize var asset **Ensure:** Initialize var devID **Ensure:** Initialize var t-sensor  **if** tx.newstate **then**    sensor.state = tx.newstate    **if** tx.enables != null **then**        sensor.enabled =tx.enabled        t-sensor = sensor    **end if**  **end if**  **return** asset.update(sensor)  **return** event.msg(’Sensor with ID ’+ devID +’ changed its state.’) = 0

**Figure 9:**
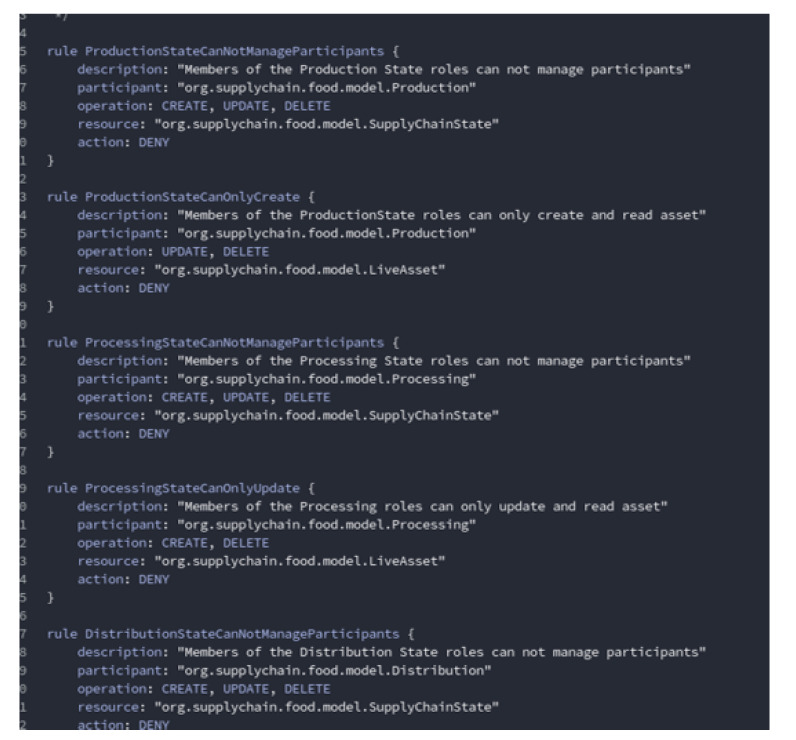
Rules implemented in a smart contract.

Figure 9 depicts a set of rules implemented in smart contracts. The network administrator can give access according to the membership of the participant and can allow a specific set of users to perform some tasks while restricting others from achieving the same task.

### 4.4. Advanced Deep Learning

We have proposed a hybrid model for the optimization of the supply chain. This model is based on a recurrent neural network (RNN), which is a class of artificial neural networks. Long short-term memory (LSTM) and gated recurrent units (GRU) are two models of RNN. Generally, the weight and bias matrixes of LSTM and GRU are random. We have used the Genetic Algorithm (GA) for the optimization of hyperparameters. GA is used to find the optimal parameters, such as the initial weights, the number of hidden layers, neurons, and epochs for neural networks [47].

**Figure 10:**
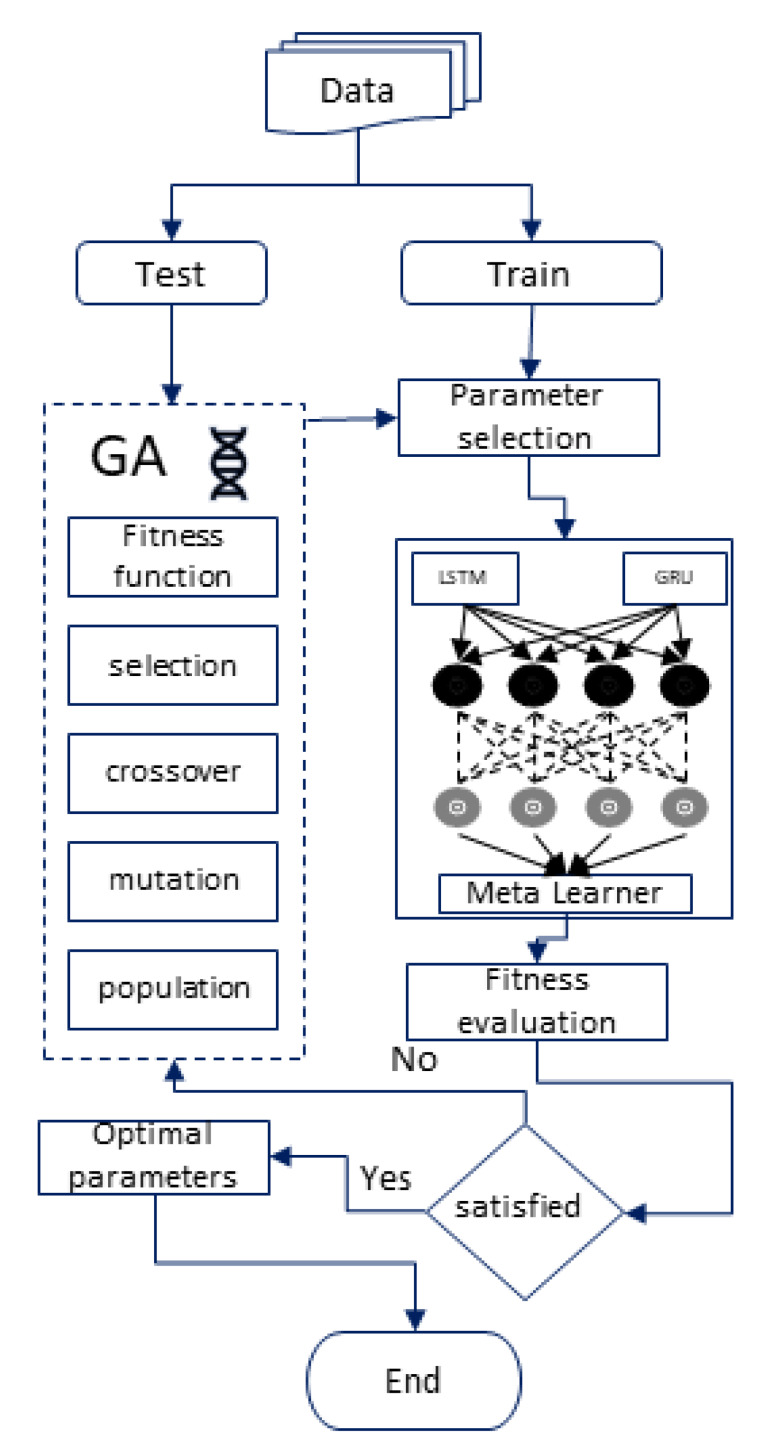
Advanced deep learning workflow.

Figure 10 shows the workflow of the proposed system. We take the data from the blockchain and divide these data into training and testing sets. Our proposed hybrid RNN takes the parameters and uses them as weights for LSTM and GRU. Both algorithms run independently on the same data; then, the prediction results of both models are used as predictors for the meta learner. Then, we evaluate its fitness. The root means square error (RMSE) of the model on the validation set will become a fitness value; if it is satisfied, then these features are taken as optimal parameters; otherwise, these features will be forwarded to the Genetic Algorithm.

### 4.5. Genetic Algorithm

The Genetic Algorithm is a heuristic search and optimization method based on the natural selection process. GAs are widely used for the optimization problem of finding approximate optimal solutions in ample parameter space [48]. The Genetic Algorithm has been widely used as the optimal parameter search technique. Wang et al. [49] used Genetic Algorithms for the modeling of power amplifier behaviors and to improve the hyperparameters of two-layer neural networks. They offered the GAs the input of hyperparameters in binary form, and the GAs treat these binary numbers as genes and train them to approach the optimal solution.

Our problem is categorized as a metaheuristics problem, for which GA, particle swarm optimization (PSO), and other evolutionary algorithms were considered. Although the difference in the performance of the PSO and GA was marginal, GA was shown to perform slightly faster [50]; therefore, we chose GA for the optimization of hyperparameters. The process of species evolution is imitated in GA, and it relies on biologically inspired functions, such as crossovers, mutation, and selection. GA works based on the natural evolution process; this is a slow, gradual process, and it makes small, slow changes in the solution in order to find the best solution. The population that is processed by GA is the number of solutions. Each solution is called an individual, and each individual solution has a chromosome. The chromosome is represented as a set of parameters that define an individual, and each chromosome contains a set of genes [51]. Besides, since it does not consider auxiliary information, GA can be used for discrete and continuous optimization. The fitness function defines the solution reserved for further replication. Crossover describes how to create a new solution from an existing solution; for example, n points cross. The mutation is used to introduce diversity and novelty into the solution pool by randomly switching or closing answers. An optimization problem that seeks to find the maximum value of the function can generally be described as per Equation (1):

(1) $$GA=\left\{\begin{array}{c} maxf\left(x\right)\\ x\;\in A\\ A\subset C \end{array}\right.$$

where *x* is the decision variable, the solution *x* that satisfies the constraints is called a feasible solution, and the set *A* represents the set of all solutions that meet the constraints *C* and is called the reasonable solution set.

### 4.6. LSTM and GRU

**Figure 11:**
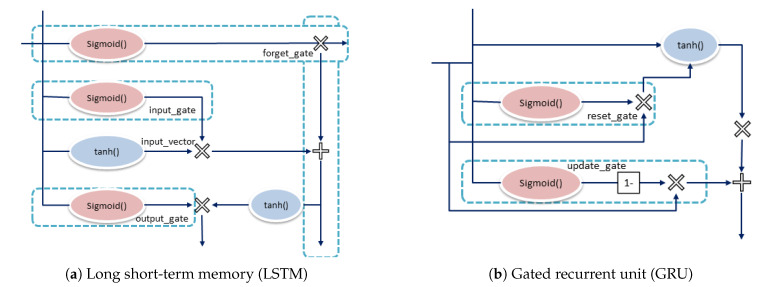
Architecture of basic LSTM and GRU units.

Long short-term memory (LSTM) and gated recurrent units (GRUs) are two widely used models of recurrent neural networks (RNNs). Figure 11 shows the architecture of basic LSTM and GRU units. RNNs have short-term memory. If the chain is long, it is not easy to transfer information from previous time steps to subsequent time steps. During back-propagation, an RNN suffers from the disappearance of values used to update the weight of the neural network, which is called a vanishing gradient problem. The problem with the vanishing gradient is that the gradient shrinks over time; if the gradient value becomes too small, no learning effect will result. Therefore, in the RNN, a layer receiving a smaller gradient update will stop learning. RNN has short-term memory as it can forget what is seen in longer sequences. Researchers present LSTM and GRU as short-term memory solutions. LSTM can learn the minimum time delay across discrete time steps by transmitting a constant error flow through a constant error carousel in a specific unit. The multiplication gate unit learns to open and close access to constant errors [52]. Researchers have used LSTM for various purposes such as topic predictions [53], price forecasting [54] and the prediction of rainfall [55].

LSTM uses memory cells to handle the long-term temporal dependencies in the data. LSTM use three types of gates: an input $\left(IG_{t}\right)$, output $\left(OG_{t}\right)$ and forget gate $\left(FG_{t}\right)$ [56]. The logistic sigmoid function usually acts as the activation function of these gates [57], and *t* is used for the current time. Figure 11a shows the structure of basic LSTM unit.

(2) $$IG_{t}=\sigma_{g}\left(W_{i}\left(h_{t-1},x_{t}\right)+b_{i}\right)$$

(3) $$OG_{t}=\sigma_{g}\left(W_{o}\left(h_{t-1},x_{t}\right)+b_{o}\right)$$

(4) $$FG_{t}=\sigma_{g}\left(W_{f}\left(h_{t-1},x\right)+b_{f}\right)$$

Equation (2) shows the function for input gate. where $W_{i}$ represents the weight for input and $b_{i}$ represents the bias vector. Equation (3) shows the function for the output gate and Equation (4) shows the function for the forget gate. $\sigma_{g}$ represents the sigmoid activation function and $h_{t-1}$ represents the hidden state as the present time in these equations.

GRU is the modified version of LSTM. It combines the two gates (input and forget) of LSTM into a single update gate, and thus has only two gates: reset $\left(RG_{t}\right)$ and update gate $\left(UG_{t}\right)$ [58]. The number of tensor operations in GRU is less than LSTM; thus, it takes less time to train. Researchers have used GRU for various purposes such as transaction fraud detection [59], prediction of sea surface temperature [60] and fault diagnosis [61]. GRU works efficiently on sequential problems.

(5) $$RG_{t}=\sigma_{g}\left(W_{r}\left(h_{t-1},x_{t}\right)+b_{r}\right)$$

(6) $$UG_{t}=\sigma_{g}\left(W_{u}\left(h_{t-1},x_{t}\right)+b_{u}\right)$$

Equations (5) and (6) show the function for the reset and update gates, respectively, where *x* denotes the input, *W* denotes the weight and *b* denotes the bias at time *t*.

### 4.7. GA-Based Hybrid Deep Learning Model

We have proposed a hybrid deep learning model that uses GA as an optimizing algorithm for feature selection. It ensemble the predictions from LSTM and GRU models.

Algorithm 2 explains the pseudo-code for the GA-based hybrid RNN model. It initializes by setting the evolution counter c = 0 and setting the maximum evolution C and randomly generates M individuals as the initial group P (0). Then, the LSTM and GRU models are trained and the fitness after ensembling them is computed. If the termination criterion is achieved, then the output of optimal parameters is given; otherwise, an increment in C is performed, and the other three functions of the GA are applied. The selection operator is applied to the group. The purpose of selection is to inherit the optimized individuals to the next generation directly or to generate new individuals through pairing and crossover to the next generation. The selection operation is based on the assessment of the fitness of the individuals in the group. After that, the crossover operator is applied to the group. The crossover operator plays a central role in Genetic Algorithms. Finally, the mutation operator is applied to the group; that is, for some individuals in the population of the string locus, a gene is used for a change in value. After the selection, crossover and mutation operations, the population P (c) obtains the next generation population P (t + 1). If c = C, the individual with the most exceptional fitness gained in the evolution process is used as the optimal solution output to terminate the calculation; this trained model will be used to forecast the supply and demand and recommend the best retailers to the end customers.

**Algorithm 2** Pseudo code for the GA-based hybrid recurrent neural network (RNN) model. Begin 
1.C = 02.initialize population P(c)3.RMSE from LSTM(c)4.RMSE from GRU(c)5.ensemble LSTM(c) & GRU(c) and compute fitness6.C = c + 17.if termination criterion achieved go to step 128.select p(c) from p(c+1)9.execute crossover p(c)10.perform mutatation p(c)11.go to step 312.output optimal parameters
 End = 0

## 5. Performance Evaluation

**Figure 12:**
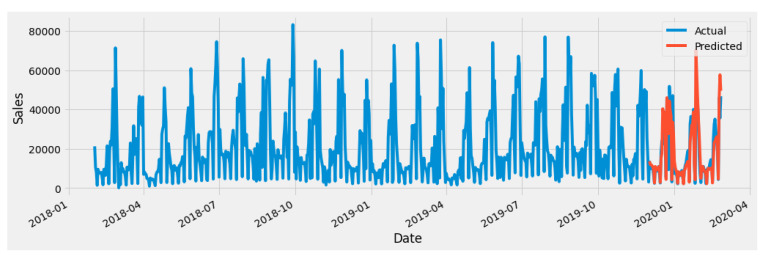
Forecasting results of sales data.

We have used an open-source dataset [62] to evaluate the performance of the proposed ADL model. The time-series data consist of daily sales data for two years from February 2018 to February 2020. We have used our proposed ADL model to understand the sales pattern for a specific store. Figure 12 shows the forecasting results of sales data, where blue lines represent the actual sales, and orange lines represent the forecasted results. The graph shows that the model predicts sales data accurately. We split the data into training and testing parts; then, we trained our model and evaluated its performance using the test data. We observed a significant improvement in the RMSE values of the proposed model. with values of 872.56, 819.65, and 512.21 with LSTM, GRU, and the introduced hybrid models, respectively. The forecasting results chart depicts actual data and prediction results as a single trending component. This demonstrates that the proposed model is robust and performs well under different seasonal effects.

We have conducted several experiments by using different performance matrices to evaluate the performance of our proposed private blockchain system. For this purpose, we used an open-source simulation tool by IBM called Hyperledger Caliper [63]. For our experimental purpose, we created three groups of 200, 400, and 800 nodes. We recorded the percentile latency (in milliseconds) of the proposed blockchain platform in the median, minimum, and maximum time.

**Figure 13:**
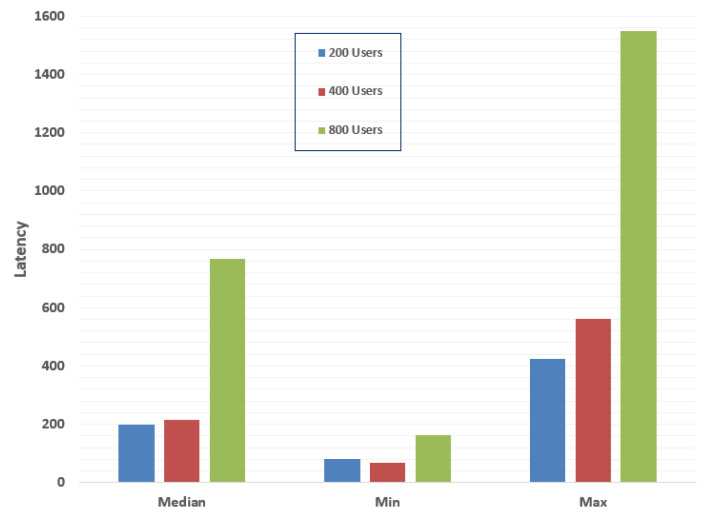
Latency for the query “get request transaction”.

Figure 13 depicts the bar graph of three groups. The chart shows that the network exhibits a change in latency with an increasing number of users; however, this will not affect the performance of the system. The median values for the group of 200, 400, and 800 users were recorded as 198, 216, and 768 ms, respectively, whereas the minimum values were 81, 69, and 164. We have observed a massive gap in the maximum amount for the group of 800 users, which was 987 ms longer than that of 400 users. Latency evaluation results indicate that with an increase in the number of users, the system exhibits an increase in the latency.

**Figure 14:**
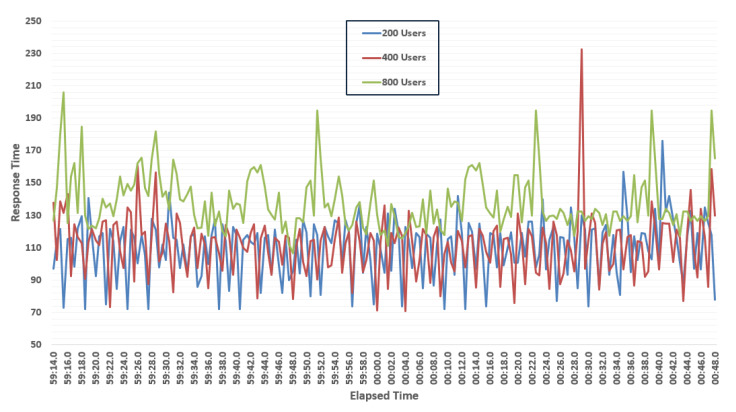
Response time for different simultaneous requests from three user groups.

We also performed simulation experiments to determine the response time of the three user groups. Figure 14 shows the response time in ms on the Y-axis and elapsed time on the X-axis. We ran simulations with a 100 millisecond granularity. In general, the response time increases with the increase in the number of users when simultaneous system requests occur. The response time evaluation results indicate that, despite the rise in the number of users, the response time remains stable. This figure illustrates that the response time is almost the same for the groups of 200 and 400 users, but as we increased it to 800, we observed a significant change in the response time. When we increased the number of users and the load on the system increased, we noted a delay in response time; however, in a private network, the number of users remains limited, and this will therefore not affect the performance of the system.

We performed the resource utilization analysis to determine the CPU and memory consumption. We also recorded the traffic in and out which was exhibited in 10 iterations by the proposed system. Table 2 shows the resource utilization analysis of the proposed method. Resource allocation does not apply to the bench client. We can observe that CA and ICA do not consume many resources in terms of memory or network. Administrator peers usually require more resources because they manage the network, and the same is true with the CouchDB, which is used as a database of the system. The traffic flow and resource utilization of the CPU and memory do not reach the higher or lower limits; thus, the network can easily handle a large number of users. The resource utilization analysis results indicate that the resource occupation rate of the proposed private blockchain network is low; due to the low usage of memory and traffic flow, it provides a comfortable user experience, and it is highly reliable.

## 6. Implications

The world population is increasing rapidly. To meet the requirements of food for this number of people, there is an urgent need for revolutionary innovations in the field of agriculture. Increasing numbers of people around the world understand the utility of the blockchain, IoT, and AI and have started to use these innovative technologies in their daily lives. Industrial IoT and advanced deep learning approaches will completely reshape the global economy; this will trigger a new agricultural revolution. The replacement of the paper tracking system means that we could have access to a tracking system with data “from farm to fork” within seconds.

The functions of the proposed management system take advantage of the IoT, blockchain, and AI technologies. It provides meat product tracking management functions with the comprehensive management of all user information. It has the option of meat product price management at all levels, including a single batch of meat product prices, as well as management and traceability information management. It offers all levels of authority management and can be set freely with authority. Blockchain and AI have proved themself to be astonishing technologies; however, some challenges need to be addressed. Both applications demand a great deal of expertise, which is difficult for small and medium-size organizations. There is a lack of awareness about the BCT, and no proper training platforms exist for educating non-specialized people. Another challenge is the lack of trust in cryptocurrencies; many countries have banned cryptocurrency on the state level. Some regulations should be made on the governmental level to allow the use of this technology in the food sector.

Many legal constraints are also involved in the implementation of such a system. Another problem is to ensure that farm owners must share the right information on the network. The involvement of governments could address these challenges. The government must take proper measures to ensure that complete data should be shared between all stakeholders across all supply chains, because if someone is not willing to be a part of this transparent chain, this could indicate nefarious motives. By implementing BCT in the meat supply chain, an environment of fair competition and a transparent market place can be created in which users can search for meat products with full confidence, and this will ensure safe and healthy food “from farm to fork”. According to Lin et al. [64], the next step in IoT-enabled e-agriculture schemes is the combination of IoT and e-agriculture with blockchain infrastructure. We can create a trusted and secure sustainable agricultural environment with the guarantee of the transparency of data by using BCT and IoT. We can engineer the monitoring of data stored in a distributed blockchain reliable using this prototype.

## 7. Conclusions

This article proposed an optimized supply chain provenance system for Industry 4.0 in the food sector using state-of-the-art technologies such as IoT, blockchain, and advanced deep learning. It provides access to the end-users to verify their food before consumption; they can check the origin and supply chain of the food on their table. Taking advantage of advanced deep learning, the participants can optimize their businesses by obtaining future forecasts and trends of food demands; they can also manage their assets on the presented immutable private ledger. Every transaction on this ledger is timestamped and encrypted, and IoT-enabled warehouses, supply trucks, and retailer shops provide the guarantee that the product was treated at the proper temperature. We have discussed the system model and architecture of the proposed system. Finally, we evaluated the performance of the proposed scheme with different scales of users. We have obtained improved results with the proposed system. We created three groups of 200, 400, and 800 users and recorded the percentile latency, response time, and resource utilization by the system. We observed that our proposed method could handle a large number of users, and that this will not affect the performance of the system. In future, this work can be enhanced by investigating more complex business networks.

## Figures and Tables

**Table 1:** Comparison of supply chain solutions. IoT: Internet of Things.

SR # [Ref]	Year	Blockchain	IoT	Deep Learning	Objective
1 [24]	2016	No	Yes	No	Tracking the environmental stress during the dairy products supply chain.
2 [25]	2017	No	Yes	Yes	Improving traceability quality using evaluation based protocols.
3 [26]	2018	No	No	Yes	Identification of missing information during the validation process of the supply chain.
4 [27]	2018	Yes	No	No	Providing sustainable environment using consortium blockchain for food trading.
5 [28]	2018	Yes	No	Yes	A credit evaluation system for accountability in the food supply.
6 [29]	2019	Yes	No	No	Using Electronic Product Code Information Services and blockchain for food safety traceability.
7 [30]	2019	Yes	Yes	No	Cost-effective and user convenient traceability system for perishable food.
8 [31]	2020	Yes	No	No	Visualizing food safety risk in the supply chain using a quantitative analysis method.
9 [32]	2020	Yes	Yes	No	Creating a value chain from farm to fork using IoT and blockchain.
10 [proposed]	-	Yes	Yes	Yes	Optimizing provenance system for food with Industry 4.0.

**Table 2:** Resource utilization analysis of the proposed system.

Type	Name	CPU	Memory	Traffic	Traffic
		(avg) MB	(avg) MB	In	Out
Process	bench-client.js	NaN	NaN	-	-
Docker	dev-peer1.war…1.0	118.09	188.2	3.0 MB	2.7 MB
Docker	dev-peer0.war…1.0	115.24	176.3	2.3 MB	1.2 MB
Docker	peer1.warehouse1.com	19.53	291.6	6.1 MB	12.4 MB
Docker	peer0.warehouse2.com	18.98	286.8	5.4 MB	10.2 MB
Docker	peer0.retailer1.com	14.16	182.2	3.5 MB	6.8 MB
Docker	peer1.retailer2.com	16.52	115.6	4.1 MB	8.3 MB
Docker	couchdb.farmer1.com	44.36	183.7	4.2 MB	8.1 MB
Docker	couchdb.farmer2.com	46.24	125.5	4.1 MB	6.3 MB
Docker	orderer.com	4.55	18.9	4.6 MB	5.8 MB
Docker	ca.Dept1	0.30	9.3	1.2 KB	1 B
Docker	ca.Dept2	0.01	8.2	0.2 KB	3.2 B

## Abbreviations

The following abbreviations are used in this manuscript:

IoT	Internet of Things
ADL	Advanced deep learning
RNN	Recurrent neural networks
LSTM	Long short-term memory
GRU	Gated recurrent units
GA	Genetic Algorithm
BCT	Blockchain technology
IIoT	Industrial Internet of Things
ICT	Information and Communications Technology
UAV	Unmanned aerial vehicles
FAO	Food and Agricultural Organization
AI	Artificial intelligence
CA	Certificate authority
RCA	Root certificate authority
ICA	Intermediate Certificate authority
CRL	Certificate revocation list
SDK	Software development kit
PoS	Point of sale
CLI	Command-line interface
RMSE	Root means square error
